# LncRNA-ANCR regulates the cell growth of osteosarcoma by interacting with EZH2 and affecting the expression of p21 and p27

**DOI:** 10.1186/s13018-017-0599-7

**Published:** 2017-07-05

**Authors:** Fei Zhang, Hao Peng

**Affiliations:** Department of Orthopaedics Surgery, Ren Min Hospital of Wuhan University, Wuhan, 430060 Hubei China

**Keywords:** LncRNA-ANCR, Osteosarcoma, EZH2, p21, p27

## Abstract

**Background:**

Osteosarcoma (OS) is one of the most common malignant tumors developed in the bone. EZH2 has been found to play pivotal roles in the development of various cancers. LncRNA-ANCR (anti-differentiation ncRNA) has been reported to interact with EZH2 and regulated osteoblast differentiation. Our study aimed to investigate the effect of lncRNA-ANCR on the tumorigenesis of osteosarcoma and explore the underlying molecular mechanism.

**Methods:**

RT-PCR was performed to detect the messenger RNA (mRNA) levels of lncRNA-ANCR, EZH2, p21, and p27 in OS tissues and cell lines. The cell proliferation, transwell invasion, and migration assays were conducted to evaluate the influence of lncRNA-ANCR depletion on the growth of OS cells. RNA pull-down assay was carried out to detect the interaction between lncRNA-ANCR and EZH2. Correlation between the expression of lncRNA-ANCR and the expression of EZH2 were analyzed by cross-tabulation.

**Results:**

LncRNA-ANCR is highly expressed in both OS tissues and cell lines. Reduced expression of lncRNA-ANCR inhibited the cell proliferation, invasion, and migration of OS cells. The cell apoptosis rate was also increased with the overexpression of lncRNA-ANCR. Mechanistically, downregulation of lncRNA-ANCR reduced the mRNA level of EZH2 and increased the expression of p21 and p27 at both mRNA and protein levels. LncRNA-ANCR interacted with EZH2 and their expression abundance was positively correlated in OS patients.

**Conclusion:**

LncRNA-ANCR inhibited the cell proliferation, migration, and invasion of OS cells possibly through interacting with EZH2 and regulating the expression of p21 and p27.

## Background

Osteosarcoma, which is also called osteogenic sarcoma (OGS), is one of the most common malignant tumors developed in bone. OS accounts for about 60% cases of all the bone cancerous tumors in both adolescence and childhood [1–3]. The patients with OS usually have a high incidence of lung metastasis, and the 5-year survival rate is less than 30% for patients suffering OS combined with lung metastasis [4–6]. Therefore, identifying the molecular targets involved in the development of OS and developing treatment strategies is quite necessary.

Long non-coding RNAs (lncRNA) are a group of non-protein coding RNAs with a length longer than 200 nucleotides [7], which is different from that of short interfering RNAs (siRNAs), microRNAs (miRNAs), Piwi-interacting RNAs (piRNAs), small nucleolar RNAs (snoRNAs), and other short RNAs. Previous study has shown that the expression levels of many lncRNAs were altered in OS tissue [8]. A recent study reported that downregulation of lncRNA TUG1 reduced the proliferation rate and increased apoptosis rate in OS cell [9], which indicated that lncRNAs may be considered as biomarkers for the diagnosis of OS and potential molecular targets for the treatment.

Enhancer of Zeste Homolog 2 (EZH2), a histone methyltransferase, has been found to play pivotal roles in the development of various cancers [10, 11]. EZH2 is highly expressed in solid tumors to initiate cell proliferation [12], and the high expression of EZH2 is negatively correlated with the patients’ outcome [13, 14]. It has been reported that overexpression of EZH2 increased the trimethylation of H3K27 on the promoters of p21, which facilitated the p53 binding on the promoter and activated the expression of p21 [15]. Additionally, p27 was also negatively regulated by EZH2 [16, 17]. These findings provided important cues to understand the molecular mechanism of EZH2 in cancer development.

LncRNA-ANCR (anti-differentiation ncRNA) has been demonstrated to be involved in regulating osteoblast differentiation [18], however, the function of lncRNA-ANCR in osteosarcoma remains largely unknown. Additionally, it has been documented that lncRNA-ANCR interacted with EZH2 to regulate the differentiation of osteoblast. In view of the function correlation of EZH2 and lncRNA-ANCR, we hypothesized that lncRNA-ANCR may play critical roles in OS.

In this study, to explore the function of lncRNA-ANCR in OS, the expression level of lncRNA-ANCR in both OS tissues and cell lines were determined. High expression abundance of lncRNA-ANCR in OS was observed. Downregulation of lncRNA-ANCR inhibited the cell proliferation of OS cells. Further investigation found that depletion of lncRNA-ANCR suppressed the expression of EZH2 and activated p21 and p27. Positive correlation between the expression of lncRNA-ANCR and EZH2 was also observed in OS patients.

## Methods

### Clinical patients and OS tissues

The tumor specimens were obtained from 20 patients with OS who underwent resection between July 2015 and March 2016 during their hospitalization in Wu Han University affiliated hospital. The tissue samples of OS patients were graded and staged by experienced pathologists after tissues were collected. According to the medical records, 14 patients have been diagnosed with cancer metastasis among the 20 OS patients. At the same time, 20 patients with lumbar discectomy underwent resection were also selected as the control group. Ethical approval was obtained by Wu Han University affiliated hospital. We had all the necessary consent from any patients involved in the study, including consent to participate in the study where appropriate.

### RT-PCR

Total RNA extraction was performed with RMeasy MiNi Kit (Qiagen, 74104, Germany). TransScript-Uni One-Step gDNA Removal and cDNA Synthesis SuperMix (ABSCI, AB452, USA) were used for reverse transcription. The RT-PCR was carried out with ABI 7500 Real-time PCR instrument and TaqMan Multiplex Master Mix (Life Technologies, 4486295, USA). GAPDH was used as an endogenous control. The following primers were used: lncRNA-ANCR-forward: 5′-GACATTTCCTGAGTCGTCTTCGAACGGAC and reverse: 5′- TAGTGCGATTTAGAGCTGTACAAGTTTC; p21-forward: 5′- TCTGGGGTVTVACTTCTTGG and reverse: 5′-ATGTGAGGAAGGCTCAGTGG; p27-forward: 5′-GATGGGGTTCACCGTGTTAG and reverse: 5′-CCCTTTCCAAACATCCATTG; EZH2-forward: 5′-TTGTTGGCGGAAGCGTGTAAAATC and reverse: 5′-TCCCTAGTCCCGCGCAATGAGC; GAPDH-forward: 5′-CGAGCCACATCGCTCAGACA and reverse: 5′-GTGGTGAAGACGCCAGTGGA. Relative RNA level was calculated using 2^ΔΔCt^ method.

### Cell culture

Human OS cell lines and osteoblast cell line hFOB1.19 were purchased from American Type Culture Collection (Rockville, MD, USA) were cultured in DMEM medium containing 10% FBS at 37 °C in a humidified atmosphere with 5% CO_2_.

### Transfection

MG-63 and UMR-106 cells were seeded into the 6-well plates with 1 × 10^5^ cells per well. Cells were cultured in DMEM medium containing 10% FBS until 70% confluence was observed. Transfection was performed with Lipofectamine 3000 (Invitrogen, L3000015, USA) according to the manufacturer’s instruction. Cells were collected after transfection for 72 h and were subjected to the following experiments. Lnc-ANCR siRNA design and preparation was performed by GenePharma Co. Ltd (Shanghai, China).

### Cell proliferation assay

Cells were seeded in 96-well plates with 3 × 10^3^ cells per well. Cell Counting Kit-8 (CCK-8) was used to detect the viability of cells according to the manufacturer’s instructions. The abundance at 450 nm was measured. The experiment was performed with three replicates.

### Western blot

Cells were lysed with the RIPA buffer and then centrifuged at 15,000 *g* for 15 min at 4 °C. The protein concentration was measured using BCA protein assay kit (Sigma, pierce23225KIT, USA). Antibodies for EZH2 (#4902), p27 (#2552), p21 (#2947), and GAPDH (#2118) were purchased from Cell Signaling Technology.

### Fluorescence-activated cell sorting (FACS) for apoptosis

3 × 10^5^ of OS cells with the indicated treatment were resuspended to make the single cell suspension. Cells were stained with Fluorescein isothiocyanate-conjugated Annexin V and 7-AAD (4ABio, FXP027-100, China). Single staining of FITC and 7-AAD was used to set the parameters and the gate. The cell apoptosis rate was detected by the flow cytometer CytoFLEX (Beckman Coulter, Inc., Brea, CA, USA).

### Cell migration and invasion assay

OS cells transfected with the indicated plasmids were collected and suspended in serum-free medium. After that, cells were added to the upper chamber covered with matrix, the lower chamber was filled with medium containing 10% FBS. After incubation at 37 °C for 24 h, cells below the membrane were fixed and stained. The numbers of migrated and invaded cells were counted under a microscope. The procedure of cell migration assay is the same to that of the invasion assay. The only difference is that common transwell chambers were used instead of matrix-coated ones.

### RNA pull-down assay

In vitro transcription of lncRNA-ANCR was performed using T7 RNA polymerase (Ambio Life). The transcription product was purified using RNeasy Plus Mini Kit (Qiagen) treatment with DNase I (Qiagen). The purified lncRNA-ANCR was then labeled with biotin using biotin RNA Labeling Mix (Ambio Life). MG-63 and UMR-106 cells were harvested and lysed with the RIPA lysis buffer. 50 μl of the lysates were aliquoted as the input, and the remaining supernatant was incubated with biotin-labeled lncRNA-ANCR at 4 °C for 2 h. Afterwards, the M-280 Streptavidin beads (Invitrogen, CA, USA) was added into the supernatant. The mixture was incubated at 4 °C for 2 h. At the same time, beads incubated directly with the supernatant of OS cells in the absence of biotin-labeled lncRNA-ANCR were used as the negative control. Western blot was performed to detect the binding between lncRNA-ANCR and EZH2. The level of lncRNA-ANCR was examined by PCR analysis.

### Statistical analyses

Data was analyzed with SPSS 19.0 software. Enumeration data were expressed as rate or percentage. Chi-square test was used for comparisons between two groups, and one-way ANOVA was used for comparisons among multiple groups. Correlations between the expression of lncRNA-ANCR and the expression of EZH2 were analyzed by cross-tabulation. *P* < 0.05 was considered to be statistically significant.

## Results

### LncRNA-ANCR was highly expressed in OS tissues and cells

**Fig. 1:**
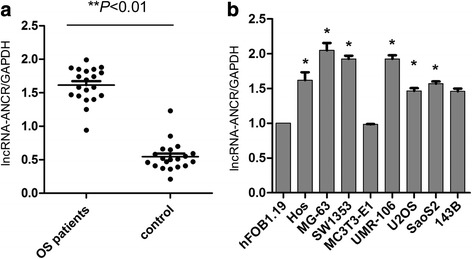
LncRNA-ANCR is highly expressed in human OS tissues and cell *lines*. **a** RT-PCT assay was performed to detect the expression of lncRNA-ANCR in OS patients and tumor-free patients. **P < 0.01. **b** The expression abundance in OS cell *lines* and osteoblast cell line hFOB1.19 were detected. *P < 0.05

RT-PCR was used to detect the expression level of lncRNA-ANCR in OS tissues and tumor-free tissues. Significant high expression level of lncRNA-ANCR was found in the OS tissues than that of the tumor-free tissues (Fig. 1a). In addition, the expression level of lncRNA-ANCR was also detected in a variety of human OS cells lines including MG-63, SW1353, U2OS, Saos2, 143B, Hos, MC3T3-E1, and UMR-106. As shown in Fig.1b, lncRNA-ANCR was highly expressed in OS cell lines in comparison with that of the osteoblast cell line hFOB1.19. These results suggested that lncRNA-ANCR was overexpressed in human OS. MG-63 and UMR-106 cell lines, which showed relatively higher expression levels of lncRNA-ANCR were selected for the following experiments.

### Downregulation of lncRNA-ANCR inhibited the cell growth of OS cells

**Fig. 2:**
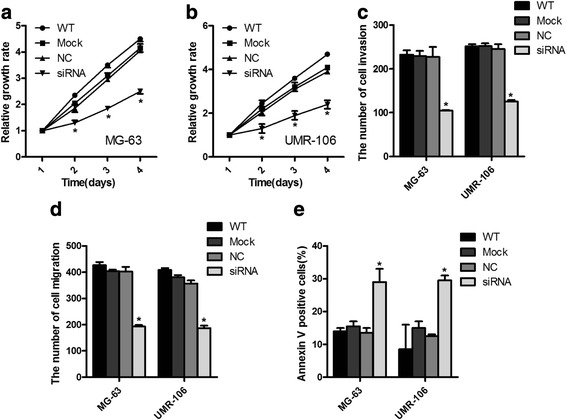
Downregulation of lncRNA-ANCR inhibited the growth of OS cells. **a**, **b** The proliferation of MG-63 (**a**) and UMR-106 cells (**b**) harboring depleted lncRNA-ANCR or control vector was detected by CCK-8 assay. *P < 0.05. **c** The data of invasion assay of MG-63 and UMR-106 cells with different treatments. *P < 0.05. **d** The migration ability of both MG-63 and UMR-106 cells with downregulation of lncRNA-ANCR or control vector was examined. *P < 0.05. **e** The apoptosis rate of MG-63 and UMR-106 cells with indicated treatments. *P < 0.05

To determine the role of lncRNA-ANCR on the tumorigenesis of OS cells, endogenous expression of lncRNA-ANCR was downregulated by transfecting lncRNA-ANCR-siRNA into OS cells. As shown in Fig. 2a, b, depletion of lncRNA-ANCR significantly inhibited the cell proliferation rate of both MG-63 and UMR-106 cells compared with that of the control cells. In addition, the transwell invasion and migration ability of OS cells harboring downregulated lncRNA-ANCR was also obviously decreased (Fig. 2c, d). Consistent with the inhibitory effect of lncRNA-ANCR depletion on OS cells, both MG-63 and UMR-106 cells expressing lncRNA-ANCR siRNA presented a significantly increased cell apoptosis rate (Fig. 2e). The results suggested that downregulation of lncRNA-ANCR negatively regulates the growth of OS cells.

### Downregulation of lncRNA-ANCR inhibited the expression of EZH2 and activated p21 and p27

**Fig. 3:**
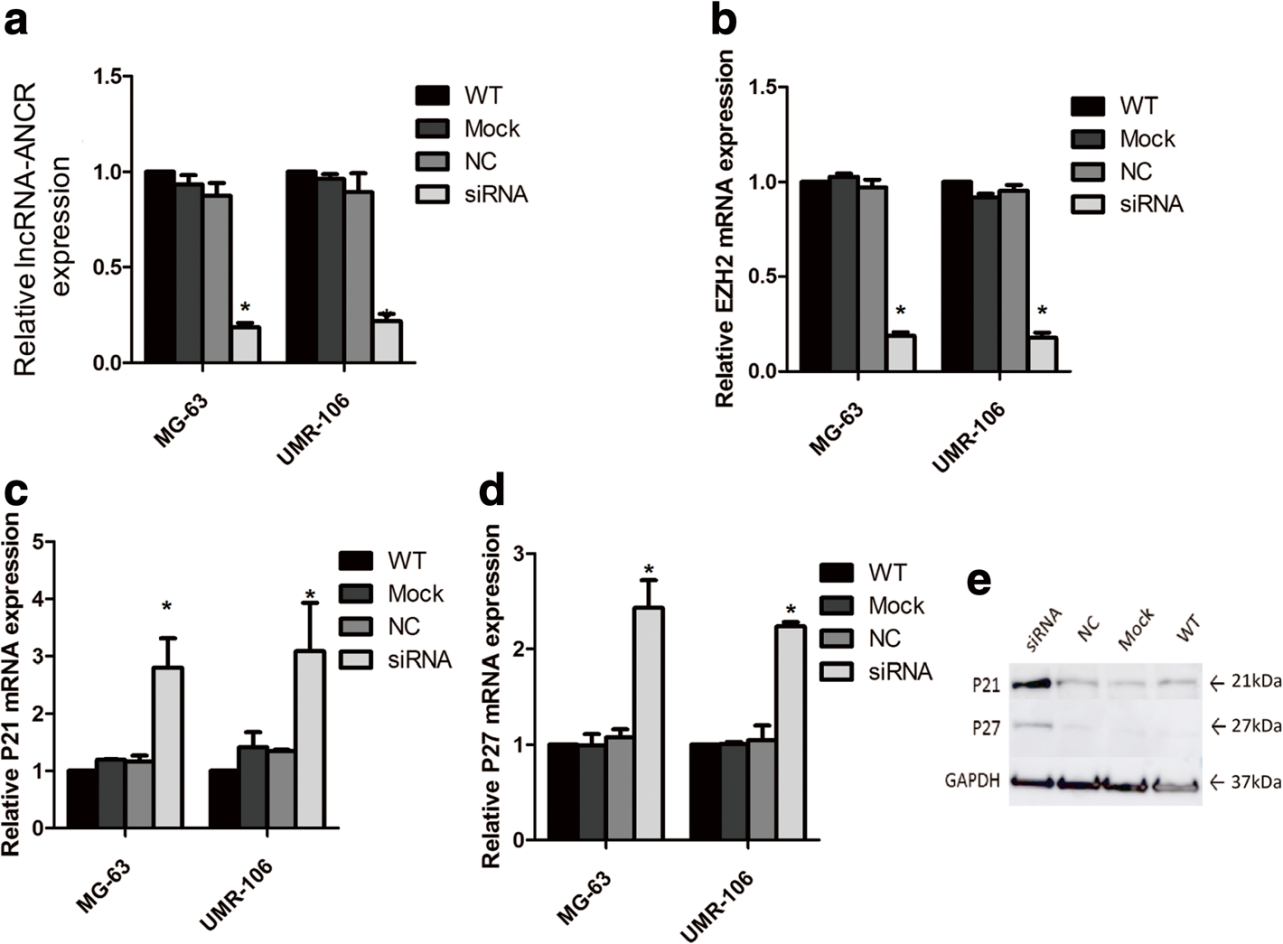
Downregulation of lncRNA-ANCR suppressed the expression of EZH2 and activated p21 and p27. **a** The downregulation of lncRNA-ANCR by siRNA interference was validated by RT-PCR. *P < 0.05. **b** The relative mRNA level of EZH2 in cells with the indicated treatments was measured by RT-PCR. *P < 0.05. **c**, **d** The mRNA level of p21 (**c**) and p27 (**d**) in MG-63 and UMR-106 cells expressing lncRNA-ANCR or control vector was detected. *P < 0.05. **e** The protein abundance of p21 and p27 in OS cells with or without lncRNA-ANCR depletion

The inhibitory effect of lncRNA-ANCR downregulation on the growth of OS cells promoted us to determine the underlying molecular mechanism. It has been reported that lncRNA-ANCR interacted with EZH2 and regulated osteoblast differentiation [18]. To detect the function of lncRNA-ANCR in OS was associated with the EZH2, the endogenous expression of lncRNA-ANCR was depleted (Fig. 3a). We firstly examined the messenger RNA (mRNA) level of EZH2 in MG-63 and UMR-106 cells harboring downregulated lncRNA-ANCR. The result showed that the mRNA abundance of EZH2 was significantly decreased in OS cells with the depletion of lncRNA-ANCR (Fig. 3b). Previous studies have reported that overexpression of EZH2 promoted cell proliferation via downregulation of tumor suppressors p21 and p27 [19, 20]. To determine the down-stream effect of the decreased EZH2 by lncRNA-ANCR knockdown, the mRNA level of both p21 and p27 was detected. As shown in Fig. 3c, d, the mRNA abundance of p21 and p27 was significantly increased in MG-63 and UMR-106 cells with the depletion of lncRNA-ANCR. Western blot analysis also showed enhanced expression level of p21 and p27 in MG-63 cells with the downregulation of lncRNA-ANCR (Fig. 3e). Similar result was also obtained in UMR-106 cells (data not shown). These data suggested that downregulation of lncRNA-ANCR inhibited the expression of EZH2 and activated the expression of p21 and p27.

### LncRNA-ANCR interacted with EZH2 and the expression of lncRNA-ANCR is positively correlated with the abundance of EZH2 in OS

**Fig. 4:**
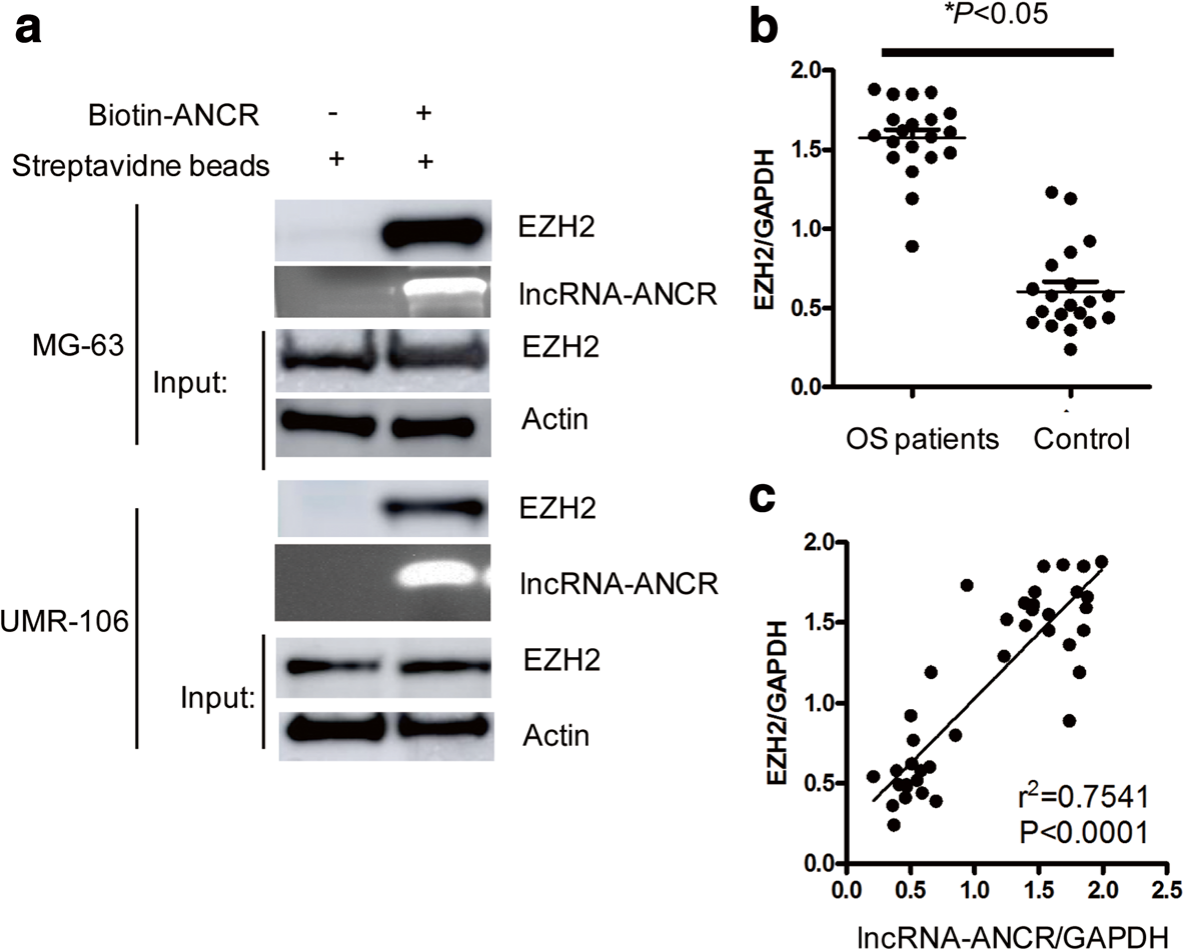
LncRNA-ANCR interacted with EZH2 and the expression of lncRNA-ANCR is positively correlated with that of EZH2. **a** RNA pull-down assay to show the interaction between lncRNA-ANCR and EZH2. **b** EZH2 is highly expressed in OS patients. *P < 0.05. **c** The expression of lncRNA-ANCR was positively correlated with that of EZH2

To further understand the molecular mechanism by which lncRNA-ANCR regulates the expression of EZH2, RNA pull-down assay was performed to detect the interaction between lncRNA-ANCR with EZH2. As shown in Fig.4a, biotin-labeled lncRNA-ANCR was incubated with the supernatant of OS cell. The binding between lncRNA-ANCR with endogenous EZH2 was detected by western blot with anti-EZH2 antibody. The result showed that lncRNA-ANCR interacted with EZH2 (Fig 4a). We hypothesized that the interaction between lncRNA-ANCR with EZH2 may trigger the regulation of lncRNA-ANCR to EZH2.

To further confirm the correlation between lncRNA-ANCR and EZH2, the expression level of EZH2 in OS patients was measured. As presented in Fig. 4b, mRNA level of EZH2 was highly expressed in OS patients. The correlation analysis between lncRNA-ANCR and EZH2 showed that the expression of lncRNA-ANCR was positively correlated with that of EZH2 (Fig. 4c, *r* ^2^ = 0.7541, *P* < 0.0001).

## Discussion

As the most common primary bone tumor in both children and adolescents, OS is a devastating disease without accurate early diagnosis and efficient treatment method, which in turn leads to the low long-term survival rate [21]. The increasing morbidity of OS has been observed during the past several decades [22]. Genetic regulation plays pivotal roles in the occurrence and development of cancer. Therefore, identifying the molecular targets involved in the occurrence and development OS will benefit the diagnosis and treatment of OS. Increasing evidence has demonstrated the involvement of lncRNA in the initiation and development of OS [8]. In our study, we found that lncRNA-ANCR was highly expressed in OS tissues and cell lines. Downregulation of lncRNA-ANCR enhanced the cell apoptosis and inhibited the proliferation, migration, and invasion of OS cells. These data indicated the oncogenetic potential of lncRNA-ANCR in OS.

Previous studies have showed that EZH2 was overexpressed in a variety of human cancers, which enhanced tumorigenesis through various signaling pathways [23]. In our study, downregulation of lncRNA-ANCR decreased the expression abundance of EZH2, suggesting that lncRNA-ANCR positively regulates the expression of EZH2. It has been reported that EZH2 promotes the cancer cell proliferation via suppressing the expression level of the cell cycle protein including p21 and p27 [19, 20]. Consistent with these finding, we found that downregulation of lncRNA-ANCR significantly increased the mRNA and protein expression of both p21 and p27. These results suggested that depletion of lncRNA-ANCR negatively regulated the EZH2-p21/p27 signaling pathway. Previous studies have shown that LncRNA-ANCR interacted with EZH2, which is important for osteoblast differentiation. Consistently, our data demonstrated the binding of lncRNA-ANCR with EZH2 in OS cells. It has been reported that overexpression of EZH2 promoted cancer cell proliferation via downregulation of tumor suppressors p21 and p27 [19, 20]. In this study, lncRNA-ANCR interacted with EZH2 and depletion of lncRNA-ANCR activated the expression of p21 and p27, we hypothesized that downregulation of lncRNA-ANCR may block the recruitment of EZH2 to the promoters of p21 and p27, which attenuates the negative regulation of EZH2 on p21 and p27. This hypothesis needs further validation.

Consistent with the high expression of lncRNA-ANCR in OS patients, overexpressed EZH2 was also found in OS patients. Correlation analyses have shown that the expression of lncRNA-ANCR is positively correlated with that of EZH2. Further investigation is required to explore the upstream regulator that controls the high expression of lncRNA-ANCR and EZH2 in OS. The overexpression of lncRNA-ANCR may be a promising target for the diagnosis and treatment in OS.

## Conclusions

LncRNA-ANCR was highly expressed in OS tissues and cells. LncRNA-ANCR depletion inhibited the proliferation, invasion, and migration of OS cells. Downregulation of lncRNA-ANCR decreased the abundance of EZH2 and activated the expression of both p21 and p27. The interaction between lncRNA-ANCR with EZH2 indicated that lncRNA-ANCR might exert its function via binding to EZH2. High expression of lncRNA-ANCR suggested its clinical significance in OS.

## Abbreviations

CCK-8: Cell counting kit-8
DMEM: Dulbecco’s modified Eagle’s medium
FACS: Fluorescence-activated cell sorting
FBS: Fetal bovine serum
lncRNA: Long non-coding RNAs
miRNAs: MicroRNAs
mRNA: Messenger RNA
OS: Osteosarcoma
PBS: Phosphate-buffered saline
piRNAs: Piwi-interacting RNAs
RT-PCR: Real-time polymerase chain reaction
siRNAs: Short interfering RNAs
snoRNAs: Small nucleolar RNAs
